# A homozygous *AHI1* gene mutation (p.Thr304AsnfsX6) in a consanguineous Moroccan family with Joubert syndrome: a case report

**DOI:** 10.1186/s13256-015-0732-3

**Published:** 2015-11-05

**Authors:** Siham Chafai-Elalaoui, Matthias Chalon, Nadia Elkhartoufi, Yamna Kriouele, Maria Mansouri, Tania Attié-Bitach, Abdelaziz Sefiani, Lekbir Baala

**Affiliations:** 1 Département de Génétique Médicale, Institut National d’Hygiène, Rabat, Maroc; 2 Centre de Génomique Humaine, Faculté de Médecine et de Pharmacie, Université Mohammed V Souissi, Rabat, Maroc; 3 Université d’Orléans & CNRS, INEM-UMR7355, Immunologie Expérimentale et Moléculaire & Neurogénétique, Orléans, France; 4 Service d’Histologie-Embryologie-Cytogénétique, Hôpital Necker-Enfants Malades, AP-HP, Paris, France; 5 Service de Pédiatrie IIA, Hôpital d’Enfants, Rabat, Maroc; 6 Université Paris Descartes, Sorbonne Paris Cité, France, Institut IMAGINE, Paris, France; 7 INSERM UMR1163, Paris Descartes – Sorbonne Paris Cité University, Imagine Institute, Paris, France; 8 Centre Hospitalier Régional d’Orléans (CHRO), Pôle de Biopathologie, Orléans, France

**Keywords:** *AHI1* mutation, Homozygosity mapping, Joubert syndrome

## Abstract

**Introduction:**

Joubert syndrome is a rare congenital disorder characterized by brain malformation, developmental delay with hypotonia, ocular motor apraxia, and breathing abnormalities. Joubert syndrome is a genetically highly heterogeneous ciliopathy disorder with 23 identified causative genes. The diagnosis is based on brain imaging showing the “molar tooth sign” with cerebellar vermis agenesis. We describe a consanguineous Moroccan family with three affected siblings (18-year-old boy, 13-year-old girl, and 10-year-old boy) showing typical signs of Joubert syndrome, and attempt to identify the underlying genetic defect in this family.

**Methods:**

We performed genome-wide homozygosity mapping using a high-resolution array followed by targeted Sanger sequencing to identify the causative gene.

**Results:**

This approach found three homozygous regions, one including the *AHI1* gene. Direct sequencing of the 26 coding exons of *AHI1* revealed a homozygous mutation (p.Thr304AsnfsX6) located in exon 7 present in the three Joubert syndrome-affected Moroccan siblings. Of more interest, this truncating mutation was previously reported in patients with compound heterozygous Joubert syndrome originating from Spain (one patient) and from the Netherlands (two patients), suggesting a possible founder effect or mutational hotspot.

**Conclusions:**

Combined homozygosity mapping and targeted sequencing allowed the rapid detection of the disease-causing mutation in the *AHI1* gene in this family affected with a highly genetically heterogeneous disorder. Carriers of the same truncating mutation (p.Thr304AsnfsX6), originating from Spain and the Netherlands, presented variable clinical characteristics, thereby corroborating the extreme heterogeneity of Joubert syndrome.

## Introduction

Joubert syndrome (JBTS; Mendelian Inheritance in Man, MIM #213300) is a rare congenital disorder characterized by a distinctive brain malformation, developmental delay with hypotonia, ocular motor apraxia, and breathing abnormalities [1]. The diagnosis is based on a characteristic feature of brain imaging, the “molar tooth sign” (MTS) reflecting agenesis of the cerebellar vermis, and brainstem alterations. The MTS has also been described in many syndromes included in the designation “Joubert syndrome and related disorders” (JSRDs) with involvement of several other organs including the eye, kidney, and liver [2–4]. JBTS is a genetically heterogeneous disorder. Thus far, 23 genes have been identified of which 22 are autosomal recessive and one is X-linked recessive.

Most genes causing JBTS encode proteins involved in primary cilia functions, thus JBTS is included in a group of disorders called ciliopathies. Mutations in several genes implicated in JBTS have been identified in other ciliopathies such as MORM syndrome (mental retardation, truncal obesity, retinal dystrophy, and micropenis; MIM #610156) [5] and nephronophthisis (NPHP; MIM #256100), Meckel syndrome (MIM #249000) [6, 7], and oral-facial-digital syndrome type 1 (OFD1; MIM #311200) or type 6 (MIM #277170), thus suggesting that various clinically distinct ciliopathies may be allelic. The considerable phenotypic overlap and wide variability of these ciliopathies can be explained by their common molecular and cellular etiology [8].

We describe here a consanguineous Moroccan family with three affected siblings (two boys and one girl) showing typical signs of JBTS with hypotonia, mental retardation, bilateral nystagmus, ataxia, retinitis pigmentosa and a MTS on brain magnetic resonance imaging (MRI). To identify the underlying genetic defect in this family, we performed a genome-wide homozygosity mapping using the high-resolution Affymetrix single nucleotide polymorphism (SNP) 6.0 array followed by direct sequencing.

## Results

### Clinical investigation

**Fig. 1:**
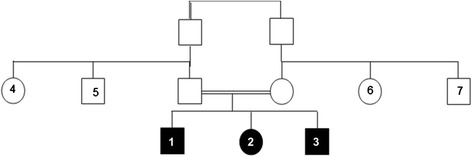
Pedigree of Moroccan family with Joubert syndrome. Patients 1, 2 and 3 were 18-, 13- and 10-years old respectively. Their parents are first cousins

We describe a Moroccan family of three affected siblings born to healthy consanguineous parents (parents are first cousins; Fig. 1).

**Fig. 2:**
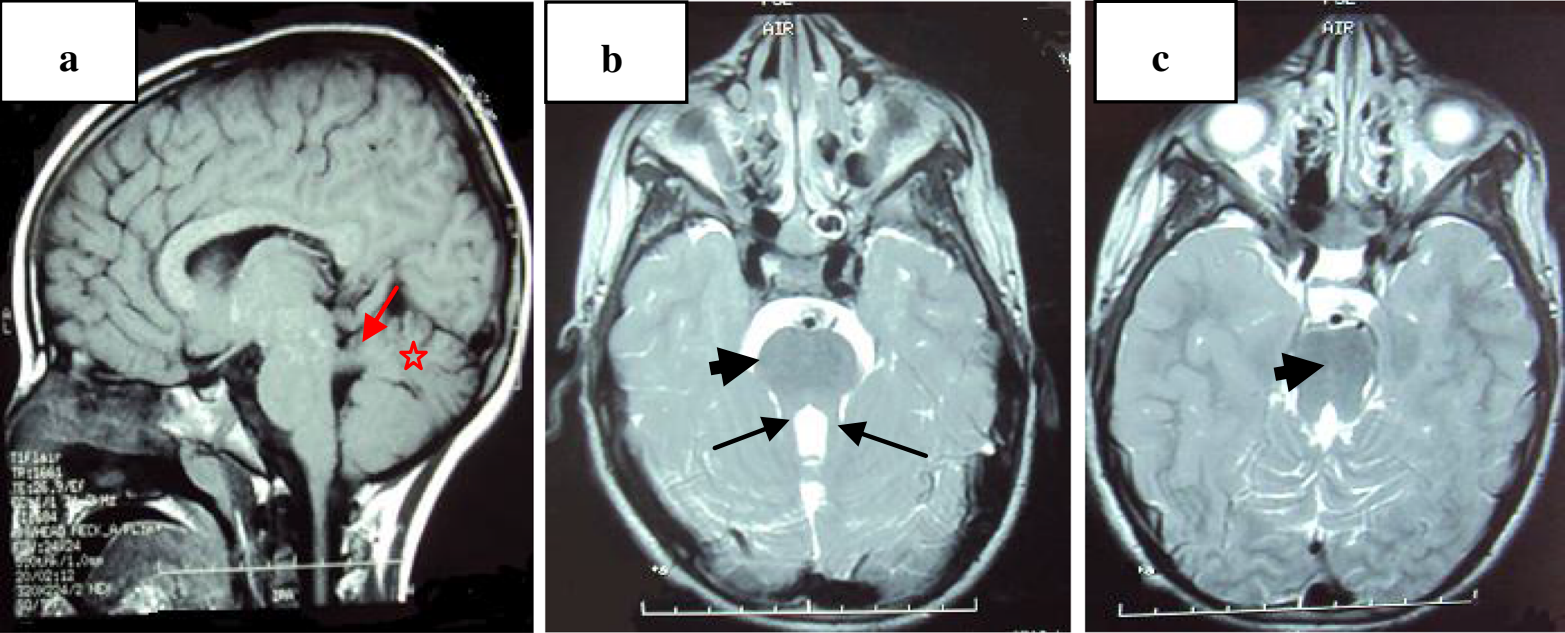
Magnetic Resonance Imaging (MRI): Sagittal and axial views through brainstem of patient 1 indicating hypoplasia of cerebellar vermis (red stars a), horizontally oriented (red arrow, **a**) and elongated (black arrows, **b**) superior cerebellar peduncles forming the “molar tooth“ sign (headarrows, **b** and **c**)

**Patient 1**, the first child, an 18-year-old boy, was referred to medical genetics for mental retardation and visual impairment. Born at term, he had neonatal hypotonia, and psychomotor delay, walked at 4 years of age and had very poor language skills. He had visual impairment, which was worse at night. He never attended school. Urea and creatinine measured in his blood were normal. On clinical examination, he had normal growth parameters, bilateral horizontal nystagmus, and ataxia. An ophthalmological examination showed retinitis pigmentosa. Brain MRI revealed a hypoplasia of his cerebellar vermis with a MTS (Fig. 2).

**Patient 2**, a 13-year-old girl, had clinical findings similar to those of her older brother with psychomotor delay and poor language skills. She walked at 2 years of age.

**Patient 3**, an 10-year-old boy, had clinical findings similar to those of his older brother and sister. He also had psychomotor delay and very poor language skills, and walked at 3 years of age.

### Molecular analysis

**Fig. 3:**
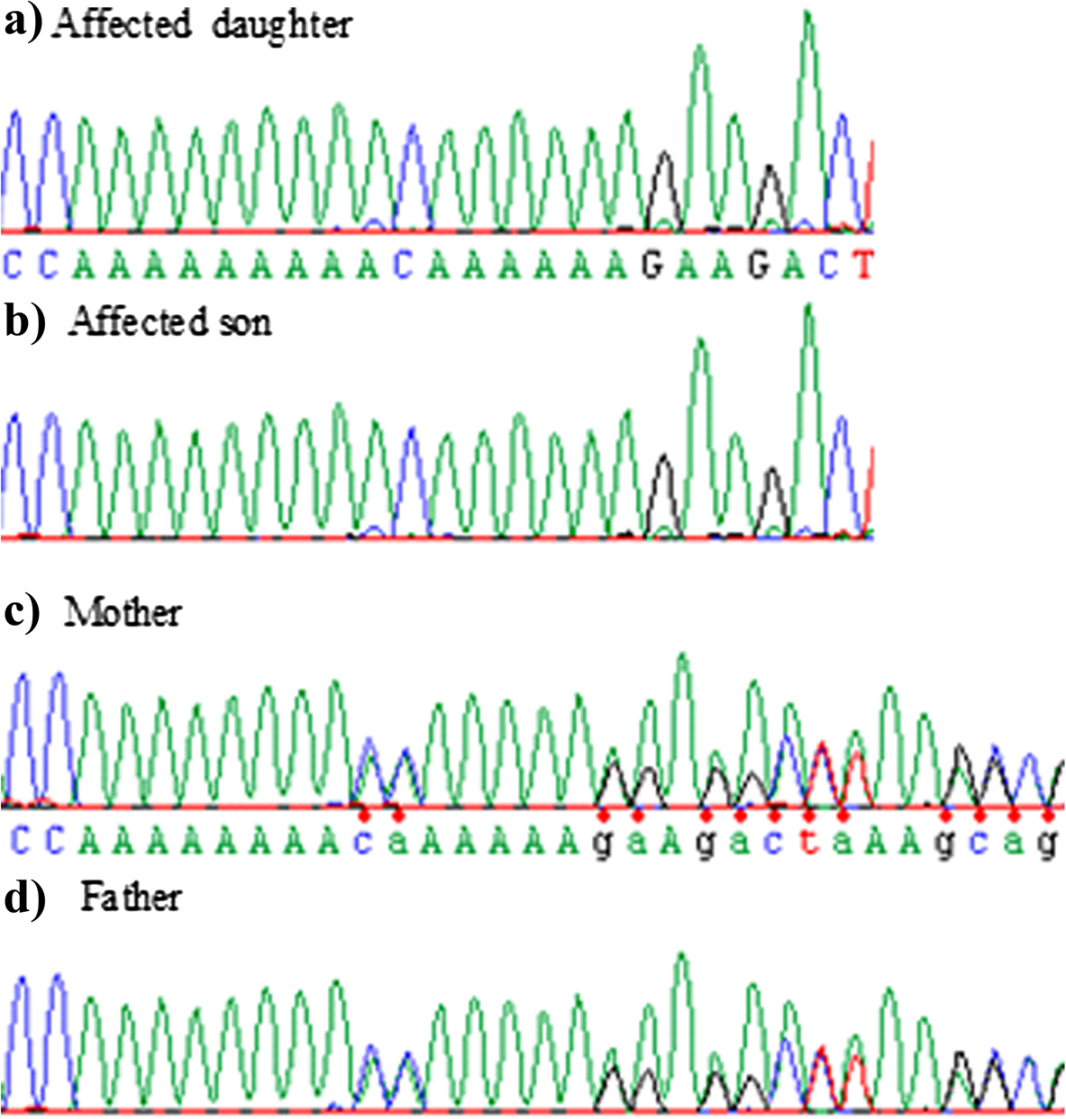
Sequence analysis. The three patients carry a homozygous mutation (p.Thr304AsnfsX6) causing frameshift and predicting a premature protein truncation (**a** : affected daughter or patient 2, **b **: affected son or patient 1). Parents are heterozygous for the mutation (**c**, **d**)

Homozygosity mapping performed using the high resolution Affymetrix SNP 6.0 array revealed three homozygous regions that were shared among affected siblings, with a size of 1.7 Mb at 5q33.3, 30.1 Mb at 14q22–24 and 28.9 Mb at 6q22.1–24 respectively. Overall, the three intervals contained more than 100 genes. We compared the detected homozygous regions with known JBTS loci, and found that the homozygous region on chromosome 6q contained *Abelson helper integration site 1* (*AHI1*), considered to be the gene at *JBST3* locus. Direct sequencing of the 26 coding exons revealed an insertion of an adenosine base (c.910dup, p.Thr304AsnfsX6), located in exon 7 (NM_017651.4), causing a frameshift and predicting a premature protein truncation. The mutation was homozygous in all patients (Fig. 3), heterozygous in their parents, and not found in controls with the same ethnic background. Otherwise, the genomic array analysis detected no pathological copy number variation (CNV) in particular at the *nephronophthisis 1* (*NPHP1*) locus.

## Discussion

The prevalence of JBTS ranges between 1:80,000 and 1:100,000 [9–11]. JBTS is genetically heterogeneous with 23 genes identified thus far. We used a whole-genome approach to identify the homozygous loci in this family, followed by a targeted Sanger sequencing of *AHI1*.

The homozygous p.Thr304AsnfsX6 mutation found in this family was reported in one affected patient from a consanguineous family originating from Spain [12] and in two patients originating from the Netherlands [11] (exon 7 for NM_017651.3). The spanish patient (family MTI-107 in the article) had compound mutations respectively in exon 8 (exon 7 in NM_017651.4) and in exon 14. The p.Thr304AsnfsX6 mutation predicts a prematurely truncated protein. Contrary to what was reported by Kroes *et al.* [11], this mutation is therefore not specific for the Dutch population.

Most *AHI1* mutations reported by Valente *et al.* [12] and Kroes *et al.* [11] are truncating, except the two V443D and R723Q missense mutations. Most mutations cluster within exons 7 to 16. The occurrence of the p.Thr304AsnfsX6 mutation in two unrelated families (Moroccan and Spanish) may be due to a founder effect, as reported for many other mutations in patients with JBTS. The c.218 G>T mutation, resulting in p.Arg73Leu in *transmembrane protein 216* (*TMEM216*) has been reported with a founder effect in the Ashkenazi Jewish population [12]. As an Arab population (Moorish or Moriscos) lived in Spain for hundreds years and many converted to Christianity, the presence of the same mutation in Arabic Moroccan and Spanish families with JBTS suggests that they might share the same founder; however, the two other patients from the Netherlands with the same (p.Thr304AsnfsX6) mutation reported by Kroes *et al.* [11] have a Dutch origin, as well as their parents, as far as several generations back (personal communication by Kroes). To investigate the genetic history of this mutation in the Moroccan, Spanish, and Dutch families with JBTS, the analysis of linkage disequilibrium (LD) between flanking polymorphic markers and AHI locus should shed light on the origin of this mutation and estimate the age of the mutation, that is, whether it was carried by a common ancestor or arose due to a mutational hotspot.

Parisi *et al.* reported that 11 % of individuals with JBTS had *AHI1* mutations [10], Kroes *et al.* revealed that 16 % of patients with JBTS from the Netherlands had mutations in *AHI1* [11], whereas Valente *et al.* identified mutations in this gene in 7.3 % of individuals in a cohort of patients with JSRDs [12]. Among this large cohort of 137 families with JSRDs and a demonstrated MTS, Valente *et al*. [12] identified 15 deleterious *AHI1* mutations in 10 families with pure JS or JS plus retinal and/or additional central nervous system abnormalities. They reported that no correlate was evident between the type of mutation (truncating, missense, or splicing) or the exon involved and the phenotypes observed [12]. They concluded that *AHI1* mutations are a frequent cause of the disease in patients with specific forms of JSRDs but are not responsible for JSRDs with liver and kidney abnormalities. Valente *et al*. [12] found that *AHI1* mutations are a frequent cause of JS with retinal involvement or other central nervous system abnormalities, or both, which is consistent with our report.

Almost 80 % of patients with *AHI1* mutations have retinal dystrophy [11, 13] and early onset congenital blindness [12]. The three patients presented here and the two patients reported by Froes *et al*. [11] carrying the p.Thr304AsnfsX6 mutation showed retinal and oculomotor abnormalities (that is, retinitis pigmentosa or Leber congenital amaurosis, nystagmus). The ocular abnormalities were not observed in the Spanish patient (MTI-107, 10-years old) reported by Valente *et al*. [12].

Renal disease consistent with NPHP has also been described in patients with JBTS with *AHI1* mutations [10, 11]. Renal ultrasound in our three patients did not reveal any abnormalities; renal disease was also absent in the Spanish and Dutch patients with the same p.Thr304AsnfsX6 mutation. However, breathing abnormalities were absent in our affected Moroccan siblings as compared to the Spanish and Dutch patients. Other neurological malformations are found to be variable among these patients with the same mutation.

JSRDs is inherited predominantly in an autosomal recessive manner. X-linked inheritance has been reported in OFD1-related JSRDs. Digenic inheritance has also been suggested. Lee *et al.* reported a digenism in JBTS, resulting from a heterozygous *CEP41* mutation in combination with either a *coiled-coil and C2* *domain containing 2A* (*CC2D2A*) or a Kinesin family member 7 (*KIF7*) mutation [14]. Such digenic inheritance has also been suggested in patients with *AHI1* mutations and *NPHP1* deletions [11]. This observation suggests that *NPHP1* deletion leads to a large range of phenotypes that can be modified by *AHI1* mutations. The CNV analysis undertaken in our patients did not show any deletion of the *NPHP1* gene.

Our study demonstrates that homozygosity mapping is a powerful method to rapidly detect the disease-causing gene especially in clinically and genetically heterogeneous disorders such as JBTS.

## Conclusions

In this study we detected a disease-causing mutation in the *AHI1* gene in a Moroccan family with JBTS by using homozygosity mapping. The rare p.Thr304AsnfsX6 mutation found in the Moroccan patients has been found in one Spanish patient and in two patients from the Netherlands in a heterozygous state suggesting that they may share the same founder ancestor. This might be more probable between Moroccan and Spanish families because of a common history. The second hypothesis proposes a mutational hotspot. In this study, the result allows accurate genetic counselling and may offer a molecular prenatal diagnosis to this family.

## Methods

### Sampling and DNA extraction

The Moroccan family was recruited in the Genetics Department of the National Institute of Health, Rabat, Morocco. DNA was extracted from whole blood using the standard protocol for the three affected patients and their parents. Written informed consent for genetic analysis was obtained from the parents for themselves and for their children included in this study, which conforms to the Helsinki Declaration and local legislation.

### Linkage analysis and homozygosity mapping

Affected individuals were selected for genome-wide SNP analysis using the high-resolution Affymetrix SNP 6.0 array containing 906,600 polymorphic SNPs and more than 946,000 unique probes for CNV detection. The genotyping of patients and parents was performed according to the manufacturer’s protocol. The regions of homozygosity (ROHs) were detected by the Affymetrix® Chromosome Analysis Suite (ChAS) software.

### Mutation analysis

Exons and intron–exon boundaries of *AHI1* were sequenced in one proband using 3130 Automated Sequencer (Applied Biosystems, Foster City, CA, USA). We used a touchdown protocol for the polymerase chain reaction (PCR) procedures to amplify all exons in the same conditions. Primer sequences and PCR conditions are available on request.

## Consent

Written informed consent was obtained from the family for publication of this case report and any accompanying images. A copy of the written consent is available for review by the Editor-in-Chief of this Journal.

## Abbreviations

*AHI1*: *Abelson helper integration site 1*
CNV: Copy number variation
JBTS: Joubert syndrome
JSRDs: Joubert syndrome and related disorders
MIM: Mendelian Inheritance in Man
MRI: Magnetic resonance imaging
MTS: Molar tooth sign
NPHP: Nephronophthisis
*NPHP1*: *Nephronophthisis 1*
OFD1: Oral-facial-digital syndrome type 1
PCR: Polymerase chain reaction
SNP: Single nucleotide polymorphism
